# Novel Intraperitoneal Treatment With Non-Thermal Plasma-Activated Medium Inhibits Metastatic Potential of Ovarian Cancer Cells

**DOI:** 10.1038/s41598-017-05620-6

**Published:** 2017-07-20

**Authors:** Kae Nakamura, Yang Peng, Fumi Utsumi, Hiromasa Tanaka, Masaaki Mizuno, Shinya Toyokuni, Masaru Hori, Fumitaka Kikkawa, Hiroaki Kajiyama

**Affiliations:** 1 Department of Obstetrics and Gynecology, Nagoya University Graduate School of Medicine, Tsurumai-cho 65, Showa-ku Nagoya, 466-8550 Japan; 2 Institute of Innovation for Future Society, Nagoya University, Furo-cho, Chikusa-ku Nagoya, 464-8603 Japan; 3 Center for Advanced Medicine and Clinical Research, Nagoya University Hospital, Tsurumai-cho 65, Showa-ku Nagoya, 466-8550 Japan; 4 Department of Pathology and Biological Responses, Nagoya University Graduate School of Medicine, Tsurumai-cho 65, Showa-ku Nagoya, 466-8550 Japan

## Abstract

Non-thermal atmospheric pressure plasma has been proposed as a new therapeutic tool for cancer treatment. Recently, plasma-activated medium (PAM) has been widely studied in various cancer types. However, there are only few reports demonstrating the anti-tumour effects of PAM in an animal model reflecting pathological conditions and the accompanying mechanism. Here we investigated the inhibitory effect of PAM on the metastasis of ovarian cancer ES2 cells *in vitro* and *in vivo*. We demonstrated that ES2 cell migration, invasion and adhesion were suppressed by PAM at a certain PAM dilution ratio, whereas cell viability remained unaffected. In an *in vivo* mouse model of intraperitoneal metastasis, PAM inhibited peritoneal dissemination of ES2 cells, resulting in prolonged survival. Moreover, we assessed the molecular mechanism and found that MMP-9 was decreased by PAM. On further investigation, we also found that PAM prevented the activation of the MAPK pathway by inhibiting the phosphorylation of JNK1/2 and p38 MAPK. These findings indicate that PAM inhibits the metastasis of ovarian cancer cells through reduction of MMP-9 secretion, which is critical for cancer cell motility. Our findings suggest that PAM intraperitoneal therapy may be a promising treatment option for ovarian cancer.

## Introduction

Ovarian cancer is considered the most malignant disease among gynaecological cancers, with over 238,700 newly diagnosed cases and 151,900 deaths worldwide every year^1^. In 2016, it was estimated that there would be 22,280 new cases of ovarian cancer and that 14,240 women would die from it^2, 3^. Because of the rapid and early metastasis to the peritoneum, almost 75% patients with ovarian cancer are initially diagnosed as having advanced-stage cancer (III and IV) and these patients have a poor prognosis with the present treatments^4^. The 5-year survival rate of patients with ovarian cancer is less than 50%^2, 5^. The current treatment for the advanced disease is debulking surgery followed by platinum-based chemotherapy via an intravenous or intraperitoneal method^6^. However, this approach is not very effective, with an overall recurrence risk of up to 30% after surgery^6, 7^.

Plasma medicine using non-equilibrium atmospheric pressure plasma (NEAPP) in the medical field is a new approach having various medical applications, such as sterilisation, blood coagulation, tissue regeneration and cancer therapy^8–10^. Many recent studies have shown that direct irradiation of NEAPP exerts anti-proliferation and apoptosis-inducing effects in melanoma, glioblastoma and ovarian cancer cells^11–16^. Besides direct plasma treatment of cancer cells, plasma-activated medium (PAM), also known as indirect plasma treatment, has been shown to have an anti-tumour effect in various types of cancers^17–27^. Few studies have reported that plasma treatment inhibited cancer cell metastasis^28–30^. If the effect of PAM against ovarian cancer metastasis is clearly elucidated, it would be a potential therapeutic strategy for not only ovarian cancer but also other types of cancers with intraperitoneal metastasis.

In our previous research, we found that PAM showed selective cytotoxicity towards cancer cells, whereas normal cells remained unaffected^17, 19, 24^. Moreover, PAM was shown to exert anti-proliferative effects in a chemo-resistant ovarian cancer cell line, which was established in our own laboratory, both *in vitro* and in *vivo* ^18^. However, considering the clinical situation, this model may not reflect the pathological conditions of ovarian cancer, with numerus micrometastatic disseminations in the peritoneal cavity. In our most recent study, we demonstrated that PAM showed more sensitivity to ovarian carcinoma cells in epithelial-mesenchymal transition^20^, which prompted further investigation of the relationship between PAM and ovarian cancer metastasis. Therefore, in the present study, we selected one of the most malignant ovarian cancer cell lines, ES2, and investigated how PAM affected cell motility. Additionally, we assessed the anti-tumour effect of PAM in a mouse model of ovarian cancer peritoneal metastasis, which might contribute to the development of a new treatment for ovarian cancer.

## Results

### PAM inhibits the viability of ovarian cancer cells, depending on the cell type, cell number and PAM dilution ratio

**Figure 1:**
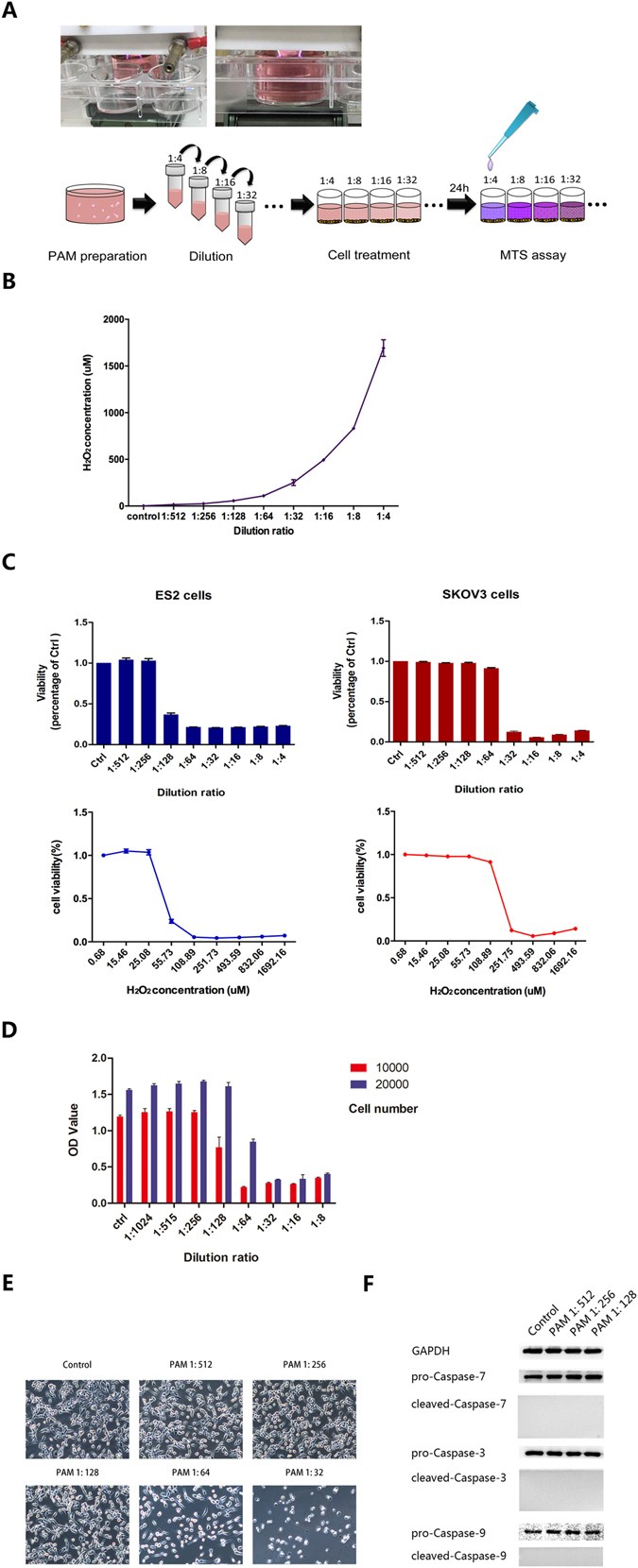
Plasma-activated medium (PAM) inhibits the viability of ovarian cancer cells, depending on the cell type, cell number and PAM dilution ratio. (**A**) The generation system of PAM and the experimental workflow. (**B**) H_2_O_2_ concentrations in each diluted PAM were measured using Amplex red reagent. (**C**) The sensitivities of ES2 and SKOV3 cells to PAM were analysed according to the LD_50_ value. (**D**) Cell viability using the MTS assay at different cell numbers and the corresponding PAM dilution ratio. (**E**) Morphological changes in ES2 cells after PAM treatment at each dilution ratio. A safe range of PAM with weak cell toxicity is confirmed. (**F**) After treatment with PAM at the safe range of the dilution ratio for 24 h, ES2 cells were collected for analysis of cell apoptosis markers using western blotting. GAPDH is used as an internal reference. The original full-length blots are presented in Supplementary Figure 3. Data from the MTS assay and H_2_O_2_ mesurement are presented as mean ± SD. Three replicates were performed.

In this study, the plasma generation system was the same as that in our previous study^12^. The brief process of original PAM generation is presented below. RPMI-1640 medium without serum was placed under the plasma head and exposed to plasma for 10 min. Owing to severe cell toxicity, we diluted the original PAM at certain ratios, and identified a ‘safe” range of diluted PAM for cancer metastasis study. After 24 h incubation with PAM, cancer cells were collected for further study (Fig. 1A). Although the exact components of plasma irradiated medium are still unclear, many researchers reported that ROS, such as hydrogen peroxide (H_2_O_2_), nitrous (NO_2_^−^) and nitrate (NO_3_^−^), had played a crucial role in the regulatory effects of plasma, such as apoptosis induction. Therefore, we investigated H_2_O_2_ as standard chemical substances in PAM and measured H_2_O_2_ concentration in each corresponding group of diluted PAM (Fig. 1B).

Initially, we selected between the two ovarian cancer cell lines ES2 and SKOV3, which could stably show tumour formation *in vivo*. At a cell number of 1 × 10^4^, the results of the viability assay indicated that ES2 was more sensitive than SKOV3 to the effects of PAM, with LD_50_ values of H_2_O_2_ concentration, 169 μM and 43 μM for SKOV3 and ES2 respectively (Fig. 1C). Therefore, we decided to use ES2 for subsequent experiments. The MTS assay was performed to check the vulnerability of ES2 to gradient ratios of diluted PAM and we found that diluted PAM at a ratio of 1:128 exerted almost no toxicity against ES2 cells. This was further confirmed by morphological findings (Fig. 1D,E). In addition, proteins of the caspase family were investigated to assess changes of cell apoptosis in ES2 cells treated with PAM, using western blotting. We found that PAM did not significantly induce apoptosis in ES2 cells at a dilution of 1:128, which we defined as the safe condition of PAM (Fig. 1F).

### PAM suppresses the migration and invasion abilities of ES2 cells

After 24 h incubation with PAM, ES2 cells were collected to investigate the metastatic capability *in vitro* through a wound-healing analysis.

**Figure 2:**
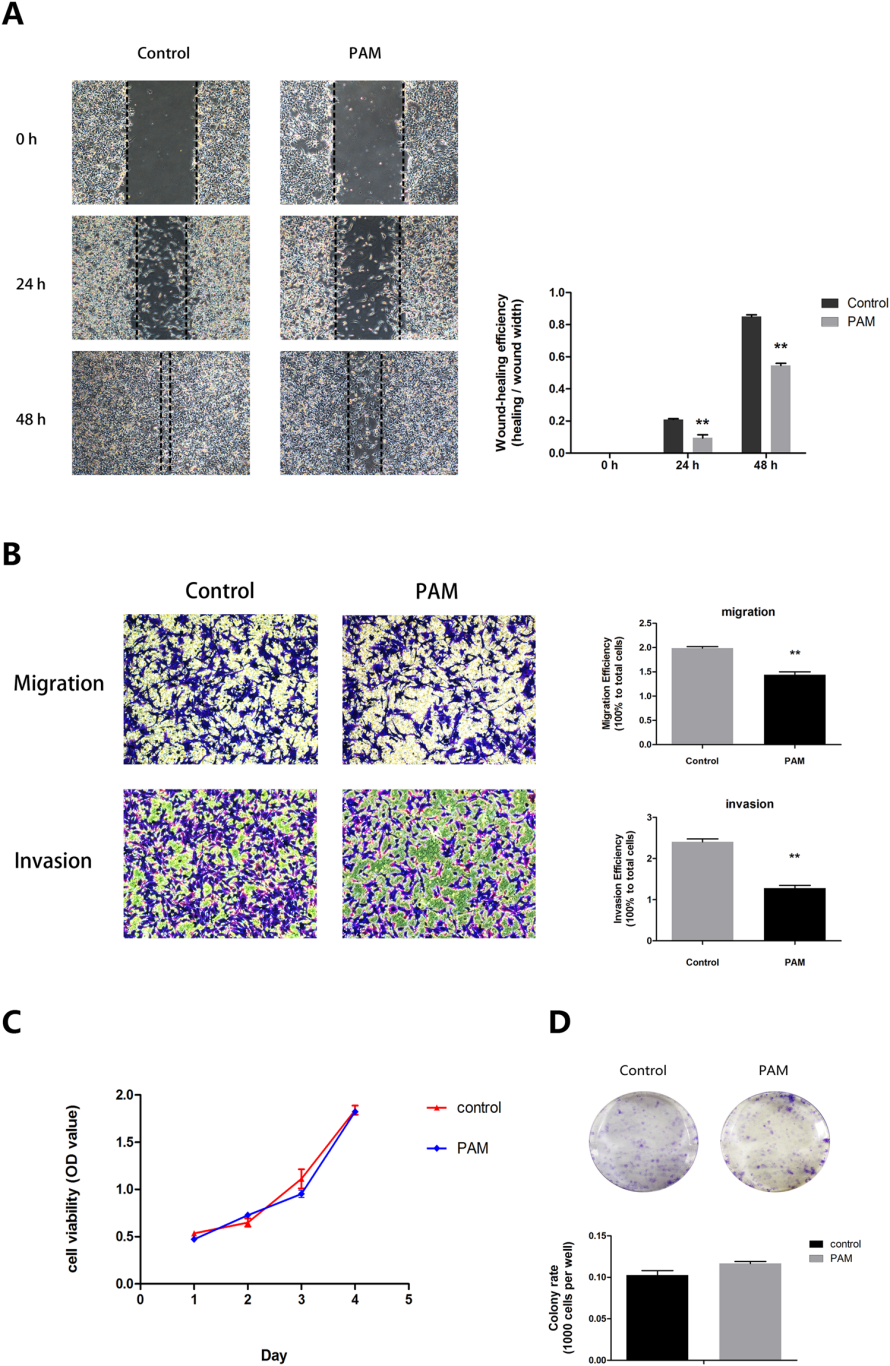
Plasma-activated medium (PAM) suppresses the migration and invasion abilities of ES2 cells. (**A**) Wound-healing assay at 0, 24 and 48 h after PAM treatment. (**B**) Transwell migration and invasion assay. Both migration and invasion abilities are calculated in comparison with the total seeded cells. (**C**) Proliferation assay for a total of 4 days. On each day, the Aqueous One Solution Cell Proliferation Assay reagent (Promega) is added for indication of cell number. (**D**) Colony-formation assay. Number of colonies was counted and compared to the total seeded cells. Three representative fields of cells in each group were captured and calculated, while one was selected for presentation in this study. Data are presented as mean ± SD. Three independent experiments with at least three replicates are performed. Statistics are shown as **P < 0.01.

Upon reaching 100% confluence, ES2 cells were wounded by using 200 µL pipette tips and exposed to PAM until analysis. PAM significantly inhibited the cell migration ability at both 24 and 48 h, when compared with controls exposed to FBS-free medium (Fig. 2A; 24 h*, P < *0.01; 48 h, *P < *0.01). Additionally, a Transwell migration assay was performed. After treatment with PAM for 24 h, cells were re-suspended and seeded into inserts of Transwell chambers. After 18 h, cells were fixed and stained for analysis. The results showed that PAM slightly inhibited the migration of ES2 cells (Fig. 2B, *P* < 0.01).

The Transwell Matrigel assay was then performed to examine the invasion ability of ES2 cells. After 24 h, it was shown that PAM significantly repressed the invasion ability of ES2 cells (Fig. 2B, *P* < 0.01).

The above results were further confirmed in a real-time cell-imaging system (IncuCyte^TM^, Essen BioScience Inc., Ann Arbor, MI, USA) for investigation of both cell migration and invasion (Supplementary Figs 1 and 2, Videos 1–6). In addition, the proliferation ability of ES2 cells assessed via the MTS cell growth assay and colony-formation assay (*P* = 0.146) remained unaffected by PAM according to our findings (Fig. 2C,D).

### PAM prevents ES2 cell plantation in co-culture with human peritoneal mesothelial cells

**Figure 3:**
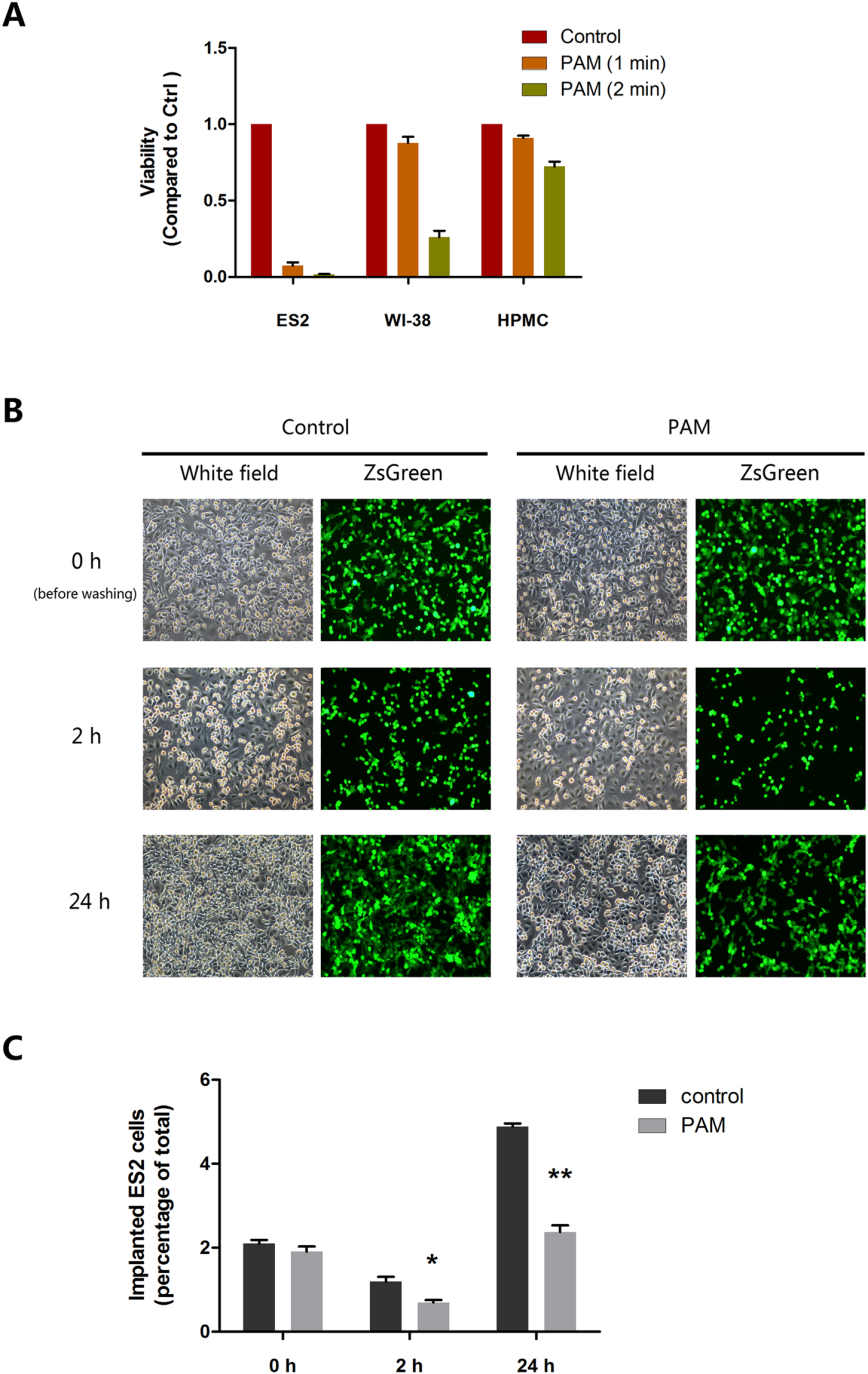
Plasma-activated medium (PAM) prevents ES2 cell plantation on co-culture with human peritoneal mesothelial cells (HPMCs). (**A**) Selective cytotoxicity of PAM against cancer cells when compared with HPMCs and lung fibroblast cells (WI-38). Cell viability was compared to that in the control group. (**B**) The co-culture model reflects the situation of peritoneal metastasis of ovarian cancer. After washing, ES2 cells (ZsGreen marked) implanted onto the monolayer of HPMCs was calculated in both the control and PAM groups. Three representative fields are captured and calculated. Data are presented as mean ± SD. Statistics are shown as *P < 0.05, **P < 0.01.

Metastasis of ovarian cancer is particularly distinguishable from that of many other types of cancers. After detaching from the primary tumour site, cancer cells adhere and implant into the peritoneal cavity and organs, which are covered by a continuous monolayer of mesothelial cells^31, 32^. In order to mimic the initial process of ovarian cancers cell metastasis, we used a co-culture model of human peritoneal mesothelial cells with ES2 cells. Primary mesothelial cells were isolated from surgical patients without diagnosis of ovarian malignancies and were seeded until 100% confluence. Then, ES2 cells with ZsGreen expressed constantly were seeded on the mesothelial cell monolayer and treated with PAM. Owing to the selective cytotoxicity of PAM, mesothelial cell viability was confirmed to be unaffected, which is consistent with our previous results^19^. Lung fibroblast WI-38 cells were selected as another group of normal cells (Fig. 3A). Within the safe range of diluted PAM, less ES2 cells adhered and implanted onto mesothelial cells continuously from 2 to 24 h after treatment (Fig. 3B; 0 h, *P* = 0.27; 2 h, *P* = 0.049; 24 h, *P* < 0.01). These results indicate that PAM prevents the initial step of the dissemination of ovarian cancer cells in the abdominal cavity.

### PAM down-regulates the expression of MMP-9 in ovarian cancer cells

**Figure 4:**
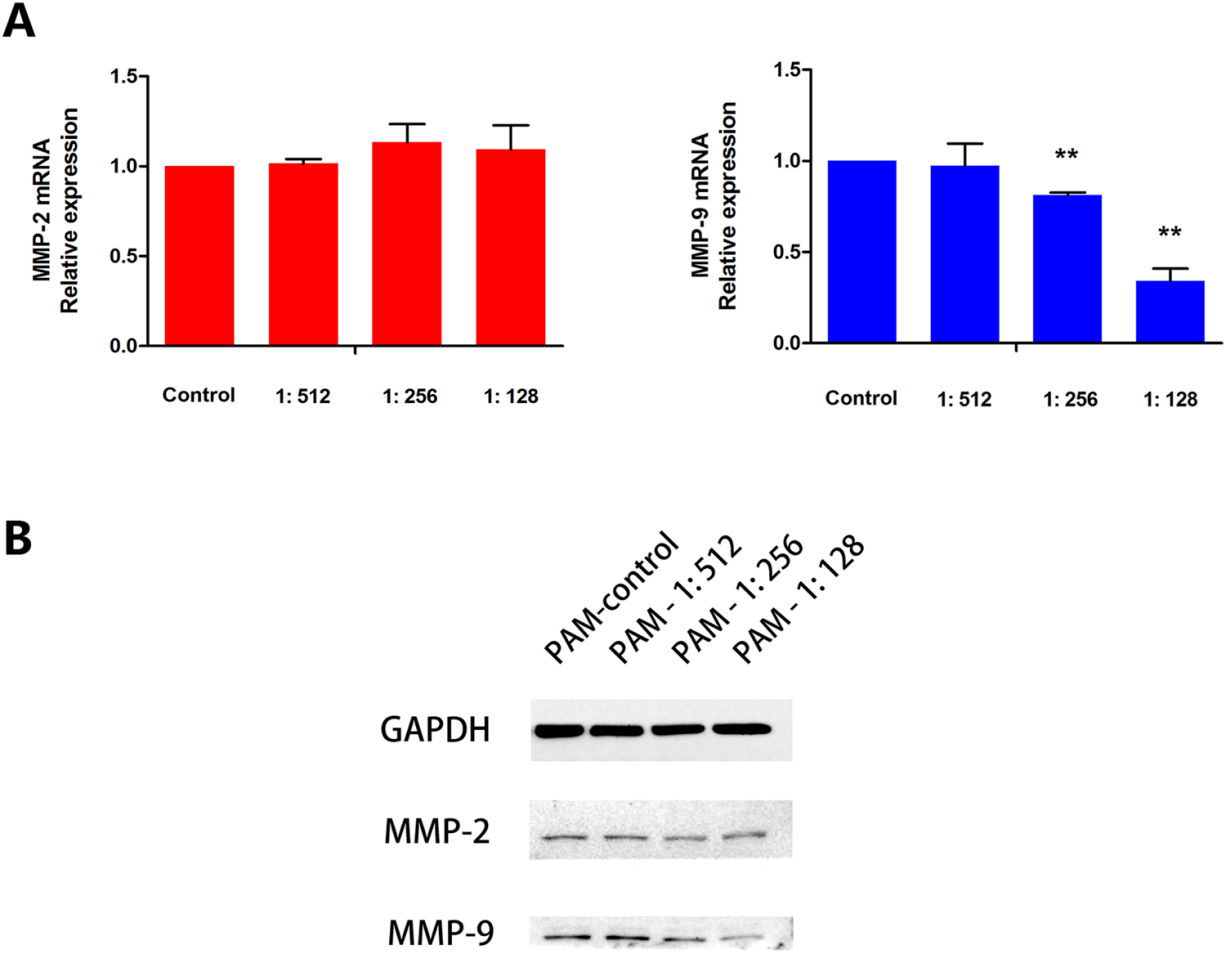
Plasma-activated medium (PAM) down-regulates the expression of MMP-9 in ovarian cancer cells. (**A**) mRNA expressions of MMP-2 and MMP-9 are calculated using real-time quantitative PCR. The MMP-2 expression is not significantly different between each diluted PAM group and the control group (*P* = 0.554, *P = *0.254 and *P* = 0.533, respectively), while MMP-9 is gradually decreased with PAM (*P* = 0.839, *P* < 0.01 and *P* < 0.01, respectively). (**B**) Protein levels of MMP-2 and MMP-9 from ES2 cells treated with PAM. GAPDH was used as the internal control. The original full-length blots are presented in Supplementary Figure 3. Data are presented as mean ± SD. Three independent experiments were performed. Each PCR reaction was performed in triplicate. Statistics are shown as *P < 0.05, **P < 0.01.

To further study how PAM inhibits the metastasis of ES2 cells, we checked the expressions of matrix metalloproteinases (MMPs), especially MMP-2 and MMP-9, which are widely reported to directly promote the invasion ability of malignant cancers^33^. We focused on the expression of MMPs in ES2 cells after treatment with PAM. Then, we further assessed the expressions of MMP-2 and MMP-9 using qPCR and western blotting. After PAM treatment for 24 h, ES2 cells were cultured with regular medium for another 24 h and then cell lysates were collected. The results from both protein and mRNA analyses showed that PAM significantly decreased MMP-9 expression, whereas MMP-2 remained unaffected (Fig. 4A,B).

### PAM inhibits MMP-9 in a MAPK pathway-dependent manner in ES2 cells

**Figure 5:**
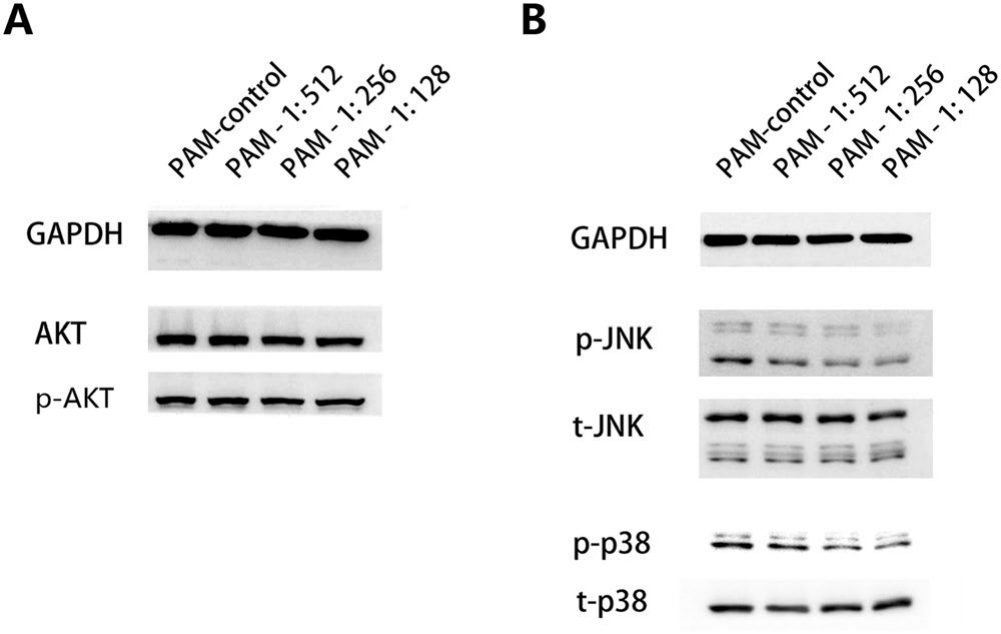
Plasma-activated medium (PAM) inhibits MMP-9 in a MAPK pathway-dependent manner in ES2 cells. After exposure to PAM (at dilution ratios of 1:512, 1:256 and 1:128, respectively) for 24 h, proteins were extracted and analysed using western blotting. (**A**) AKT pathway regulation. Both phosphorylated AKT and total AKT expression were investigated. (**B**) MAPK pathway regulation in ES2 cells with PAM treatment. The two canonical key regulators JNK1/2 and p38 were investigated at both the phosphorylation and total levels. GAPDH was selected as the internal reference. The original full-length blots are presented in Supplementary Figure 3. Three independent experiments were performed for each of the western blotting assays.

To further elucidate the mechanism associated with the regulation of metastasis by PAM, we investigated the activity of the upstream signalling pathways AKT and MAPK, in which ERK1/2, JNK1/2and p38 act as key regulators. Both the MAPK and AKT pathways are broadly reported to directly or indirectly regulate MMP-2 and MMP-9, as well as tumour migration and invasion. With regard to both phosphorylation and total level of AKT, the results showed that PAM did not affect the AKT pathway under our conditions (Fig. 5A). However, PAM significantly prevented the phosphorylation of JNK1/2 and p38 MAPK and slightly inhibited the phosphorylation of ERK1/2 (data not shown), although it did not decrease total expression (Fig. 5B). These results indicate that PAM inhibits ES2 cell metastasis by suppressing MMP-9, through MAPK inhibition.

### Anti-metastatic effect of PAM is antagonized by the ROS scavenger

**Figure 6:**
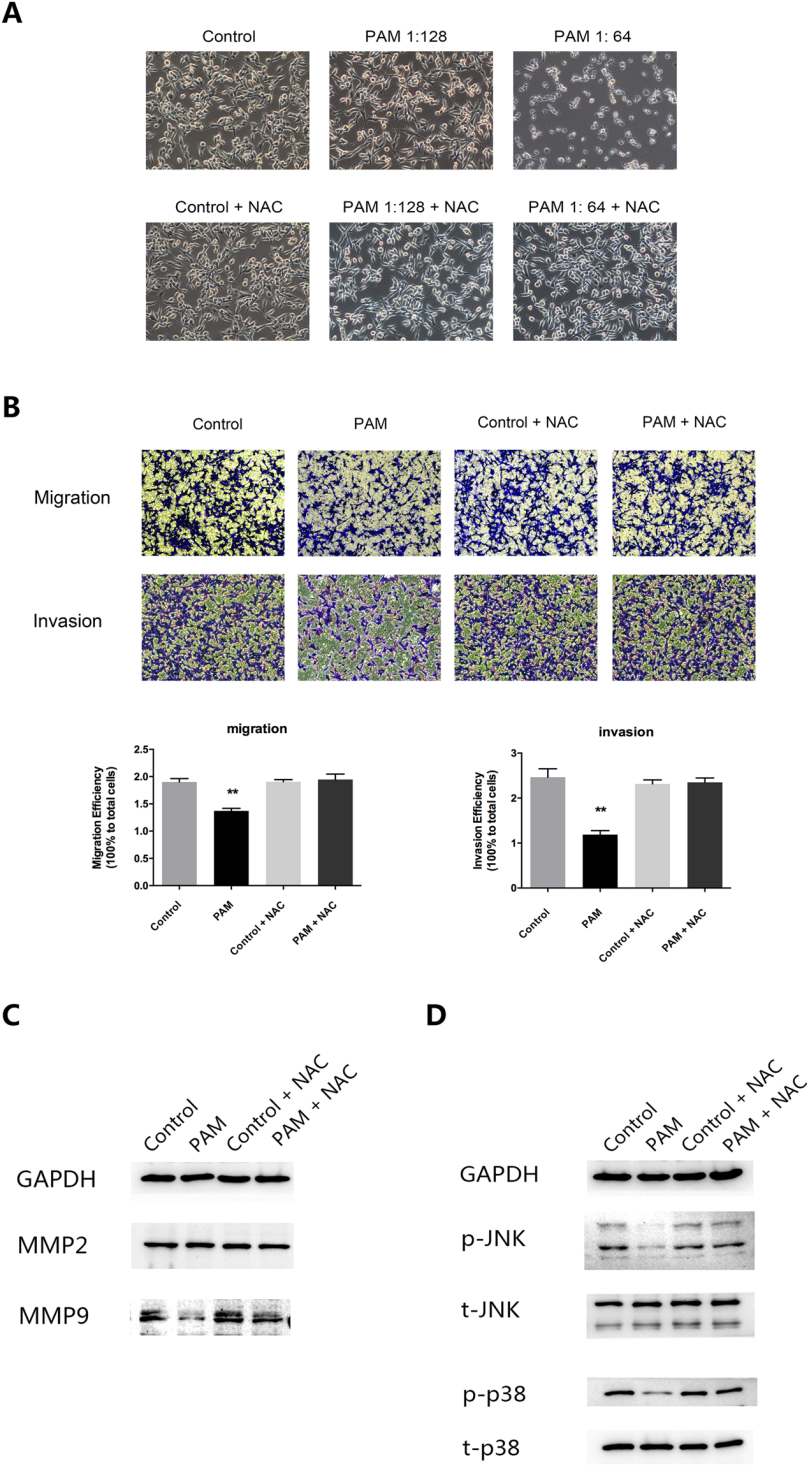
Anti-metastatic effect of PAM is antagonized by the ROS scavenger. Diluted PAM wsa incubated with NAC (4 mM, Sigma-Aldrich, St. Louis, MO, USA) at room temperature for 30 min before the following treatments. (**A**) Morphological changes of ES2 cells after PAM treatment together with or without NAC. (**B**) Cell migration and invasion analysis of ES2 cells by transwell assay. Both migration and invasion ability were calculated in comparison to the total seeded cells. (**C**) Protein expressions of MMP-9 and MMP-2 in ES2 cells after PAM with or without NAC treatment. (**D**) Protein expressions of both phosphorylated and total level of MAPK pathway components, JNK1/2 and p38. GAPDH was chosen as an internal reference. The original full-length blots are presented in Supplementary Figure 3. In transwell assay, three representative fields of cells in each group were captured and calculated, while one was chosen for presentation in this study. Data were presented as means ± SD. Three independent experiments were performed. Statistics were shown as *P < 0.05; **P < 0.01.

It has been reported that ROS plays a crucial role in anti-tumor effects of plasma. N-acetylcysteine (NAC) was used as an antioxidant broadly. Thus, in order to investigate whether ROS plays a functional role in anti-metastatic effect of PAM in ovarian cancer cells, we performed rescue experiments by PAM incubated with NAC for 30 min at room temperature before treatment. The addition of NAC showed no effect to the morphologic changes of ES2 cells, however, in the group of stronger PAM, anti-tumor effect of PAM was rescued by NAC (Fig. 6A). This is also in accordance with our previous conclusion that ROS is one of the effectors in PAM to induce cell apoptosis^18, 19^. In our transwell assays for both cell migration and invasion, the results showed that PAM (diluted at 1: 128) significantly reduced migrated and invaded ES2 cells (Fig. 6B, *P* < 0.01), however, the reduction effect was nearly completely countervailed by NAC (Fig. 6B, *P* = 0.756 and *P* = 0.822, respectively). As found in this study, PAM down-regulates the expression of MMP-9 (Fig. 5A) and affects the MAPK pathway by inhibiting phosphorylation of JNK1/2 and p38 (Fig. 5B). So we further performed western blotting to check the role of ROS in mechanism regulation. The results showed that NAC inhibited the down-regulation of MMP-9 by PAM, while MMP2 remained unaffected by PAM or NAC (Fig. 6C). Furthermore, we found that NAC antagonized the inhibitory effect of PAM on phosphorylation of JNK1/2 and p38, although the total level of JNK1/2 and p38 were unchanged (Fig. 6D). Taken together, these results indicate that anti-metastatic effect of PAM on ES2 cells might be, at least partially, attributed to ROS.

### PAM treatment prolonged longevity in an ovarian cancer mouse model

**Figure 7:**
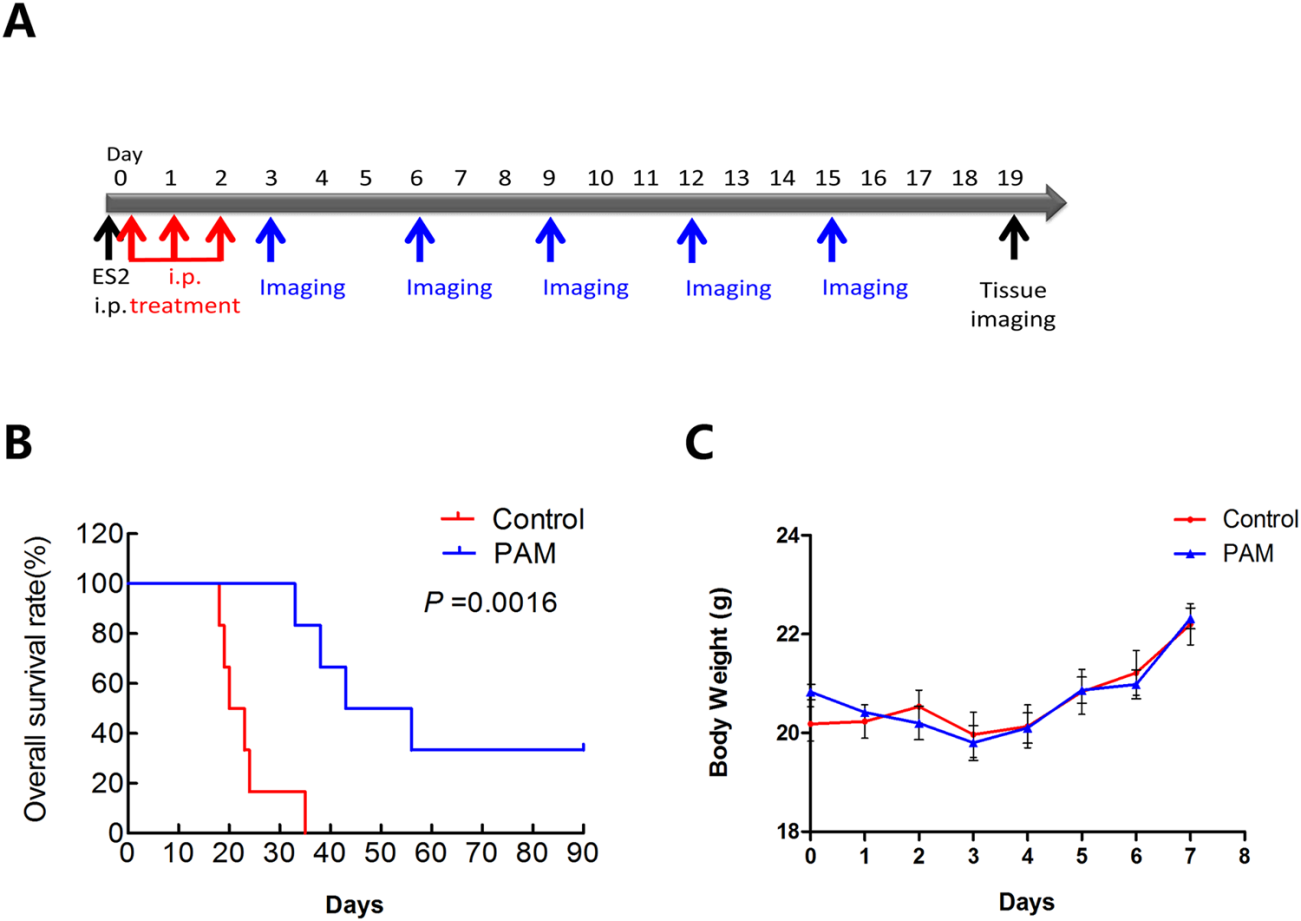
Plasma-activated medium (PAM) treatment prolongs longevity in an ovarian cancer mouse model. (**A**) The treatment and investigation manual of PAM therapy (intraperitoneal) *in vivo*. (**B**) Survival analysis of mice using the Kaplan–Meier method in the PAM therapy and control groups (n = 6). The analysis considers a total of 90 days. (**C**) Body weight comparison between each group of mice.

To examine whether PAM therapy affects survival, a mouse model of intraperitoneal injection was used. Six-week-old female BALB/c nude mice (n = 6) were intraperitoneally injected with 1 × 10^6^ ES2 cells. Intraperitoneal PAM injection was started 15 min after ES2 cell injection on the same day and was subsequently performed once a day for a total of 3 days (Fig. 7A). Survival analysis was performed using the Kaplan–Meier method; the survival rates were poorer in the control group than in the PAM therapy group (Fig. 7B, *P* < 0.01). However, body weight was not significantly different between the two groups (Fig. 7C).

### PAM suppresses intraperitoneal metastasis in a mouse model

**Figure 8:**
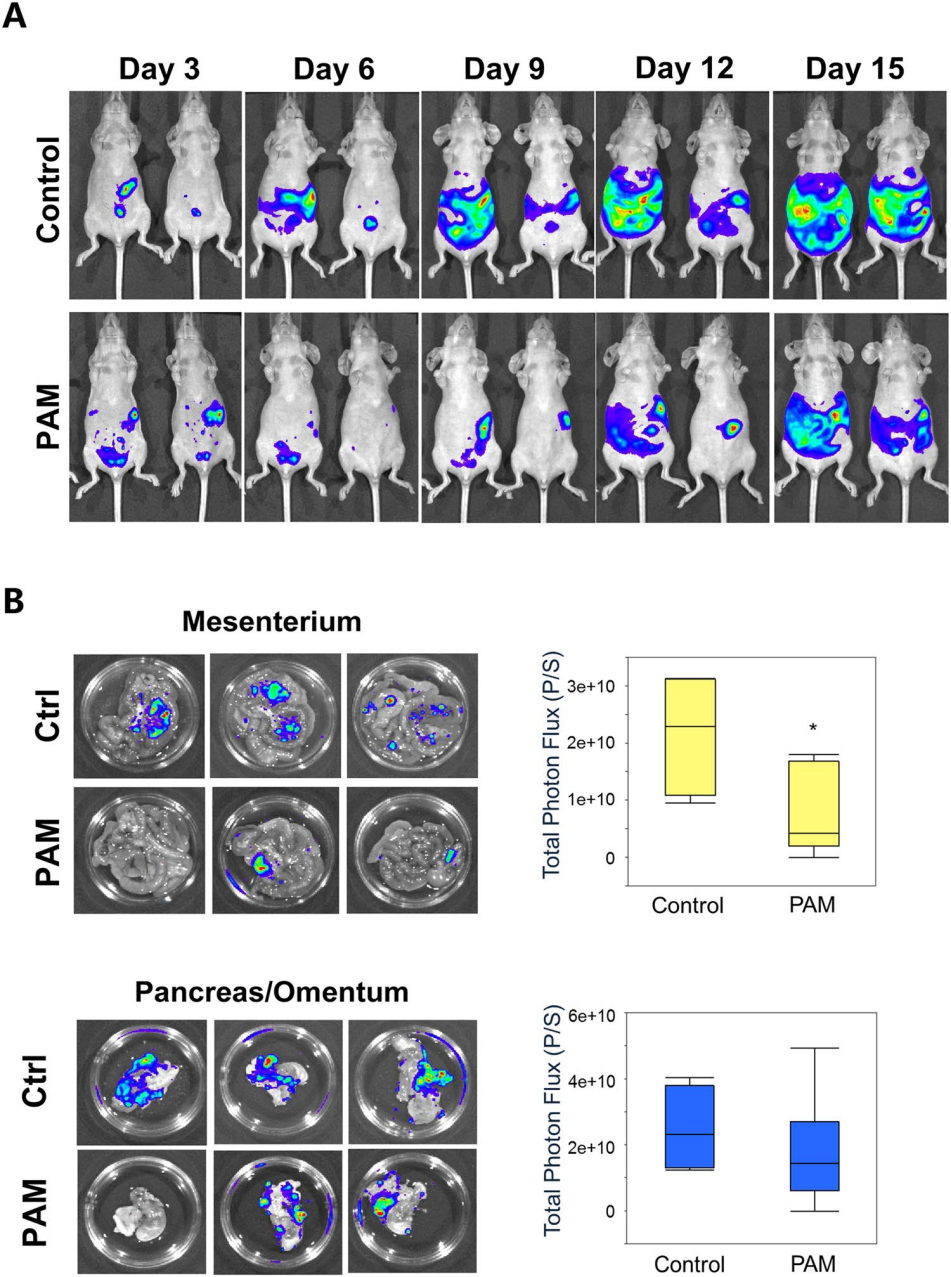
Plasma-activated medium (PAM) suppresses intraperitoneal metastasis in an ovarian cancer mouse model. (**A**) Peritoneal metastases between the PAM and control groups are indicated with bioluminescence using the IVIS system every 3 days, until day 15. (**B**) On day 19, metastasis of ES2 cells onto peritoneal tissues (mesenterium and omentum/pancreas) is investigated and quantified with bioluminescence (photon/s) using the IVIS system. Data are presented as mean ± SD. Statistics are shown as *P < 0.05.

To examine whether PAM therapy inhibits intraperitoneal dissemination in a mouse model, an *in vivo* imaging technique was used. Mice (n = 6) were intraperitoneally injected with 1 × 10^6^ ES2 cells, which stably expressed luciferase. PAM treatment was the same as that in the survival tests. Based on the bioluminescence value from luciferase-expressing ES2 cells, the peritoneal cancer metastasis state was monitored using the IVIS 200 Imaging System (Caliper Life Science, Hopkinton, MA, USA) every 3 days. The results showed that PAM significantly inhibited intraperitoneal metastasis of ES2 cells (Fig. 8A). After sacrifice, peritoneal metastatic organs were examined and assessed using the IVIS 200 Imaging System. We demonstrated that mesenteric metastasis was significantly inhibited by PAM, although the omentum showed no obvious recovery (Fig. 8B,C, *P* = 0.044 and *P* = 0.500, respectively). These results confirm the anti-metastatic effect of PAM against ES2 cells *in vivo*.

## Discussion

Non-thermal plasma therapy has been widely considered as an applicable but challenging approach for next-generation cancer therapy. The cost and tissue damage might be lower with non-thermal plasma therapy than with conventional cancer therapy. When directly exposed to plasma, cancer cells in a culture dish undergo anti-tumour processes in a distance-dependent manner from just below the plasma^12, 34^. We have previously demonstrated the site-specific and superficial effects of plasma in rat liver *ex vivo* ^35^. These finding suggested that the target of direct plasma treatment was restricted to superficial lesions and not disseminated, invasive cancer cells, which were typical characteristics of ovarian cancer. More recently, PAM has emerged as a new branch of plasma treatment involving indirect plasma therapy, which may be more feasible than the direct plasma therapy because cancer cells inside the peritoneal cavity are directly and completely exposed to cytotoxic liquids.

For debulked ovarian cancer, the intraperitoneal delivery of chemotherapeutics such as cisplatin and paclitaxel is considered clinically advantageous^36^. However, intraperitoneal chemotherapy is criticised mainly because of its severe toxic effects, as well as subsequent peritoneal complications such as abdominal pain^37^. In our previous research, PAM was found to exert selective toxicity against cancer cells^17, 19, 24^. In this study, we demonstrated that primary peritoneal mesothelial cells were not affected by PAM when compared with ES2 cells. Moreover, in the *in vivo* study, PAM intraperitoneal injection exerted little influence on body weight. In this regard, PAM intraperitoneal therapy could be a safe and practical option for ovarian cancer treatment.

On the other hand, thus far, only few studies have reported the effect of plasma on cancer metastasis. Li *et al*. found that direct plasma treatment inhibited cervical cancer cell invasiveness^29^; therefore, we were interested in whether plasma, especially PAM, might have an effect on ovarian cancer cell metastasis. We demonstrated that safe ranges of PAM, which presented weak or even no toxicity to cancer cells, inhibited ES2 cell invasion, migration and implantation onto a monolayer of mesothelial cells. Moreover, this study provided more profound evidence of the efficiency of PAM treatment in an *in vivo* model given that the results showed that PAM significantly prevented ovarian cancer cell metastasis in the abdominal cavity (Fig. 7A–C). In addition, Kaplan–Meier survival analysis revealed that mice from the PAM-treatment group had better survival predictions. Considering the current situation of temporary intraperitoneal chemotherapy, PAM could be an alternative method alone or together with other chemo-drugs for metastatic ovarian cancers.

MMP family members, especially MMP-2 and MMP-9, are known to digest collagen, gelatin and other components of the extracellular matrix (ECM), resulting in the breakdown of the barriers of cancer cells. Degradation of the ECM by these proteolytic enzymes is an essential step in cancer cell invasion and metastasis^33, 38^. This enables cancer cells to invade neighbouring tissues or even move to distant organs. Therefore, the production of MMP-2/9 is thought to directly promote invasion and migration of cancer cells. By suppressing MMPs mRNA and protein expressions, cancer metastasis might be prevented. It was reported that direct plasma treatment suppressed MMPs expression in benign melanocytic tumours^39^. Our results showed that PAM inhibited invasion and migration by repressing MMP-9 expression at both the transcriptional and post-transcriptional levels. However, MMP-2 was independent of PAM regulation in this study.

The expression and activity of MMP-9/2 were reported to be greatly dependent on the MAPK-signalling pathway, including the key regulators of ERK1/2, JNK1/2 and p38^40^. Phosphorylation of ERK, JNK, or p38, mediated by various inflammatory cytokines and growth factors, activates the MAPK pathway. Suppression of any of these components has been reported to decrease the expression of MMP-9/2 and potentially prevent cancer cell migration and invasion^41–43^. In this study, PAM greatly inhibited the phosphorylation of JNK1/2 and p38, but there was less suppression of the phosphorylation of ERK. Additionally, many studies have frequently reported that the AKT pathway is actively involved in the regulation of MMP-9, as well as of cancer metastasis^44, 45^. Our previous study demonstrated that PAM down-regulated the phosphorylation of AKT and ERK in glioblastoma under the condition of cell death induction^17, 27^. However, in this study, PAM showed no significant inhibitory effect on the AKT pathway. We further investigated whether any other signalling pathways played a major role in the regulation of MMP-9 by PAM. The results demonstrated that PAM limitedly regulated the MAPK pathway by inhibiting phosphorylated JNK1/2 and p38. The associations of PAM with these cellular factors and pathways remain unclear. Plasma consists of highly reactive components, including electrons, ions, radicals and UV, and it interacts with surrounding molecules such as moisture and oxygen and nitrogen in air, resulting in secondary highly reactive components^46^. Based on many plasma studies, it is well known that the biological effects of PAM are responsible for ROS/RNS species such as H_2_O_2_, NO_2_^−^ and NO_3_^−^, generated by reactive species in a gas phase and liquid phase and a direct interaction of plasma with liquid. ROS/RNS in a liquid can influence the cell surface or gain entry into the cell, inducing biological changes such as alteration of signal transduction and gene expression, which can elicit cell death and growth, as well as cellular morphological changes. It is hypothesised that the anti-tumour effect of plasma depends on the production of ROS. Many recent studies revealed that ROS is also related to the regulation of cancer metastasis by causing loss of cell adhesion or inhibiting cell migration and invasion^47^. Additionally, Zong *et al*. clearly demonstrated that ROS prevented cell invasion in pancreatic cancer by inhibiting the ERK/MMPs pathways^48^. A previous study also reported that ROS attenuated the AKT pathway in T-cells^49^. In this study, we also confirmed that ROS in PAM could suppress cancer cell migration and invasion through inhibiting the MAPK/MMPs pathways. Therefore, the anti-metastasis effect of PAM on ovarian cancer cells might be partially attributed to the ROS/RNS generated from plasma. However, further studies should be performed to investigate the functional and active particles in the whole system of medium stimulated by plasma.

**Figure 9:**
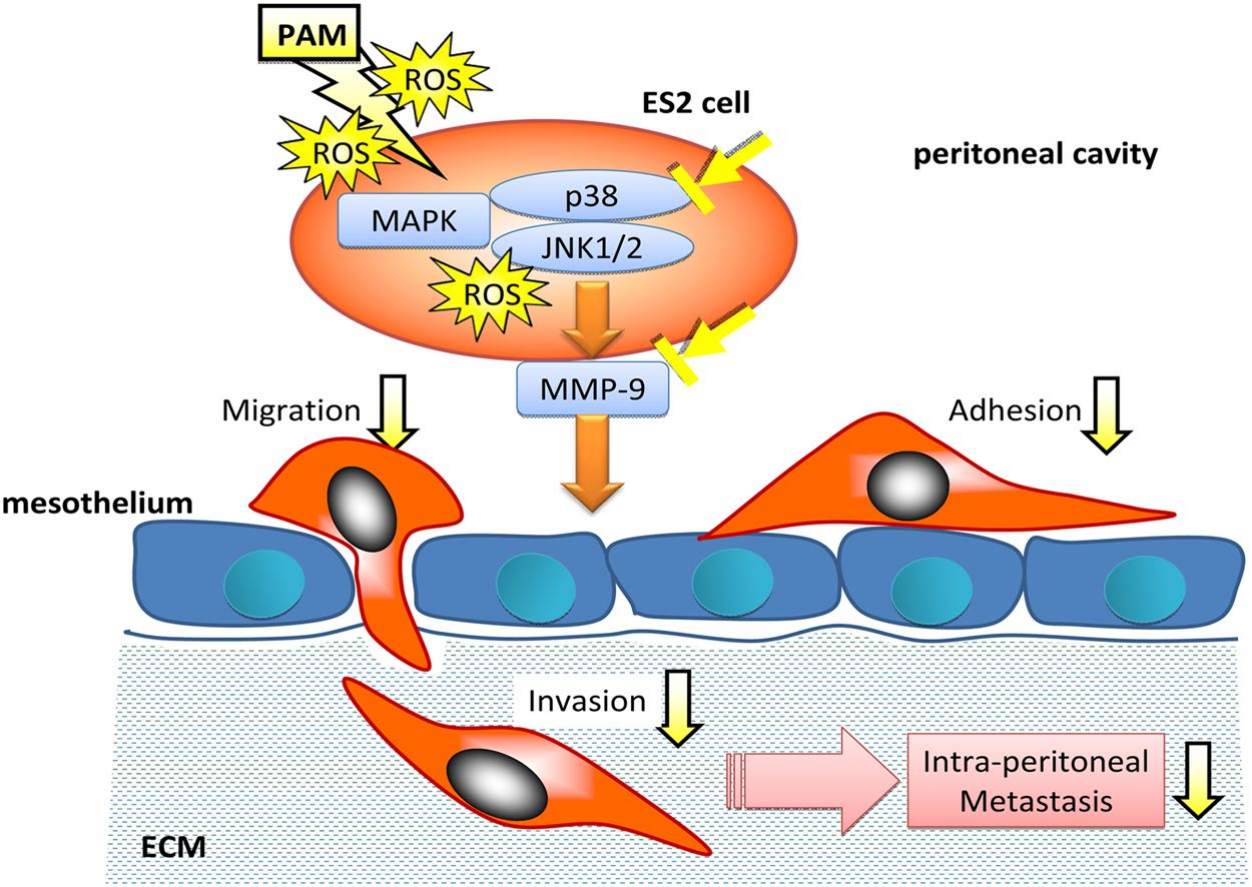
Mechanisms of anti-metastatic effect of PAM. ROS in PAM diffuses into ES2 cells and down-regulats MMP-9 expression via inhibiting of MAPK pathway, supressing cancer cell adhesion, migration and invasion onto mesothelial cells lining the peritoneal cavity. Finally, PAM prevents intraperitoneal metastasis.

In conclusion, our results demonstrated that PAM inhibited ovarian cancer ES2 cell metastasis both *in vitro* and *in vivo*, resulting in prolonged survival in a mouse model. This inhibition was associated with a decrease in MMP-9 expression, which was dependent on the attenuation of the phosphorylation of JNK1/2 and p38 MAPK (Fig. 9). Our findings might help in the development of a new clinical strategy for ovarian cancer therapy involving the use of PAM via intraperitoneal administration.

## Materials and Methods

### Cells

ES2, SKOV3 and WI-38 cell lines obtained from the American Type Culture Collection (ATCC, Manassas, VA, USA) were maintained in RPMI-1640 medium (no. R8758, Sigma-Aldrich, St. Louis, MO, USA) with 10% FBS and penicillin/streptomycin. Human primary mesothelial cells (HPMCs) were isolated from the omentum of patients undergoing surgery and were cultured under the same condition^50^. This study was approved by the institutional ethics committee of Nagoya University (approval number: 1234). Stable cell lines that expressed ZsGreen or luciferase were generated using a recombinant retrovirus following a previously described method^51^. In brief, 293 T cells were transfected with the pQCXIP vector encoding each gene as well as with the pVPack-GP and pVPack-Ampho vectors for the production of retrovirus particles (Agilent Technologies, Santa Clara, CA, USA). The culture supernatant was collected 48 h later and applied to cells with 2 mg/mL polybrene (Sigma-Aldrich). Cells were cultured for 24 h and then 1 mg/mL puromycin (Sigma-Aldrich) was added to select the infected cells.

### Experimental plasma system and preparation of PAM

We used the NEAPP system as the plasma-producing device. The details of this experimental NEAPP system have been previously described^12, 52, 53^. In brief, discharge conditions were in argon gas (2 standard litres/min; slm) excited by applying 10 kV of a 60-Hz commercial power supply to two electrodes 8 mm apart. We exposed the above NEAPP to RPMI-1640 (no. R8758, Sigma-Aldrich) without FBS separately from the cells, which was designated as PAM. The separation distance between the plasma source and the medium surface (L) is critical to consistently reproduce data and, therefore, all experiments were performed under a set condition (L = 3 mm). The duration of the plasma treatment was 10 min. In a 12-well plate, 5.5 mL of RPMI-1640 medium was placed and was treated with NEAPP at the centre of each well.

### Cell viability assay

Cells were seeded into 96-well plates. On the following day, cells were treated with the appropriately diluted PAM for 24 h. Cell viability was assayed using the Aqueous One Solution Cell Proliferation Assay kit (Promega, Madison, WI, USA), according to the manufacturer’s instructions. Absorbance was then measured at 490 nm using a microplate reader (ELx808; BioTek Instruments Japan, Tokyo, Japan).

### Measurement of Hydrogen peroxide concentration

The Amplex red hydrogen peroxide/peroxidase assay kit (Thermo Fisher Scientific, Waltham, MA, USA) was used for H_2_O_2_ measurement according with manufacturer’s instructions. Amplex can be converted to resorufin, a highly fluorescent compound upon oxidation by H_2_O_2_ in the presence of horseradish peroxidase (HRP). Fluorescence was detected using fluorescence microplate reader, Mithras LB 940 (Berthold technologies, Wildbad, Germany) setting at the excitation/emission wavelength filter, 530/600. Fluorescence intensity of Amplex was converted into absolute H_2_O_2_ concentrations (μM) by a H_2_O_2_ standard curve within the linear region.

### Western blotting

After treatment with PAM for 24 h, cells were collected by using RIPA lysis buffer (Millipore, Temecula, CA, USA) with protease inhibitor cocktail tablets (Roche Diagnostics, Indianapolis, IN, USA), and proteins were extracted according to the manufacturer’s instructions. Proteins were separated using 10% SDS polyacrylamide gels in an electrophoresis system (Bio-Rad, Hercules, CA, USA). The proteins were then transferred onto PVDF membranes (EMD Millipore, Billerica, MA, USA) at a current of 250 mA for 2 h. After blocking in 5% non-fat milk for another 2 h, the PVDF membranes were incubated with relevant primary antibodies at 4 °C overnight. On the second day, the PVDF membranes were incubated with HRP-conjugated secondary antibodies at room temperature for 1 h. After washing, membranes were reacted with ECL^TM^ Western Blotting Detection Reagents (GE Healthcare, Backinghamshire, UK) in order to detect the target proteins. The protein bands were examined using the ImageQuant LAS 4000 mini system (GE Healthcare).

### RNA isolation and qPCR

RNAiso Plus (TaKaRa Bio Inc., Kusatsu, Japan) was used to extract total RNA from cells, according to the manufacturer’s instructions. Reverse transcription was performed by using ReverTra Ace ^®^ RT Master Mix (TOYOBO, Osaka, Japan) to synthesise cDNA at 37 °C for 15 min. Quantitative PCR was conducted using LightCycler^®^480 SYBR Green I Master (Roche Diagnostics) and monitored in real-time using the LightCycler^®^480 PCR system (Roche Diagnostics) with the method of 2^−ΔΔ^CT. Expressions of all target genes were normalised to GAPDH as reference. Primers used in this study were as follows: MMP-2 forward: 5′-CAGCCCTGCAAGTTTCCATTC-3′; MMP-2 reverse: 5′-CTTCTTGTCGCGGTCGTAGTC-3′; MMP-9 forward: 5′-ACGCAGACATCGTCATCCAGT-3′; MMP-9 reverse: 5′-GGACCACAACTCGTCATCGTC-3′; GAPDH forward: 5′-CAAGGCTGAGAACGGGAAG-3′; GAPDH reverse: 5′-TGAAGACGCCAGTGGACTC-3′. All PCR reactions were performed in triplicate.

### Proliferation and colony-formation assay

After treatment with PAM for 24 h, cells were collected and re-suspended. They were then seeded into 96-well plates at 1,000 cells/well. Cell viability was determined using the Aqueous One Solution Cell Proliferation Assay kit (Promega) every day for a total of 4 days. OD values at a wavelength of 490 nm were measured using a microplate reader (ELx808; BioTek Instruments).

For colony-formation assay, cells were seeded into 6-well plates at a concentration of 1,000 cells/well. After 7 days, cells were fixed with 4% paraformaldehyde and stained with 0.5% crystal violet. Colony numbers were counted using Image J (National Institute of Health, Bethesda, MD, USA).

### Wound-healing assay

Cells were seeded into 6-well plates until 100% confluence. Then, linear wounds of equal width were created by using 200 µL pipette tips. The culture medium was changed to PAM or to serum-free medium for control. Migrated cells were observed and photographed at 24 and 48 h, respectively. Three representative fields were selected and the wound-healing ability was calculated as follows: migrated distance/wounded distance. Each distance was measured using Image J.

### Transwell migration and invasion assays

For this assay, 2 × 10^5^ cells were seeded into each well of 6-well plates until the next day. The culture medium was changed to PAM for 24 h. Then, cells were re-suspended in FBS-free medium for the Transwell assay. CORNING^®^ co-star^®^ Transwell Permeable 5-mm inserts (8.0-μm polycarbonate membrane; Corning, Corning, NY, USA) were used for the migration analysis. A total of 4 × 10^4^ cells were added into each Transwell insert and incubated for 18 h. For the Matrigel-invasion assay, CORNING^®^ Matrigel^®^ Invasion chambers (8.0 μm; Corning) were used and a total of 4 × 10^4^ cells were added into each upper chamber and cultured for 24 h. The lower chambers included 10% FBS medium. Cells that did not migrate or invade in the upper chambers were removed using cotton sticks. Finally, the upper chambers were fixed in 4% paraformaldehyde and stained with 0.5% crystal violet. Three representative fields of migrated or invaded cells were selected and counted using Image J.

### *In vivo* study

All procedures involving mice and experimental protocols were approved by the Animal Experimental Committee of the Graduate School of Medicine, Nagoya University (permission no. 28268). The animal study was carried out in accordance with the Guidelines for Animal Experiments of the Nagoya University School of Medicine. Six-week-old female nude mice (BALB/C) (n = 6) were obtained from Charles River Laboratories Japan, Inc. (Yokohama, Japan). A total of 1 × 10^6^ ES2 cells were suspended in 300 μL of PBS and intraperitoneally injected. Intraperitoneal PAM injection was administered 15 min after ES2 cell injection on the same day and was subsequently administered once a day for a total of 3 days. Anaesthetised mice were injected with 75 mg/kg D-luciferin (Caliper Life Sciences, Hopkinton, MA, USA) to acquire images using the Xenogen IVIS 200 Imaging System (Caliper Life Sciences). Analysis was performed using the Living Image software (Caliper Life Science) by measuring the photon flux of the isolated tissues and the abdominal area of the mice in the supine position.

### Statistical analysis

All data are expressed as mean ± SD. The statistical significance of differences was analysed using the Student’s *t*-test. A *P*-value < 0.05 was considered to indicate statistical significance. Kaplan–Meier survival analysis was performed using JMP software (SAS Institute Japan, Tokyo, Japan).

### Data availability

The datasets supporting the finding of this study are available within the article and its Supplementary Information files, or from the corresponding author upon reasonable request.

## Electronic supplementary material

Supplementary information

Video 1

Video 2

Video 3

Video 4

Video 5

Video 6
