# Supplementary material for: Novel Intraperitoneal Treatment With Non-Thermal Plasma-Activated Medium Inhibits Metastatic Potential of Ovarian Cancer Cells
Source: Sci Rep. 2017 Jul 20;7:6085. doi: 10.1038/s41598-017-05620-6 (PMC5519696; doi:10.1038/s41598-017-05620-6)
Supplement: Supplementary file 1 — Supplementary information [file 41598_2017_5620_MOESM1_ESM.pdf]

Supplementary information

NOVEL INTRAPERITONEAL TREATMENT WITH NON-THERMAL PLASMA-ACTIVATED MEDIUM INHIBITS METASTATIC POTENTIAL OF OVARIAN CANCER CELLS

Kae Nakamura[1]†, Yang Peng [1]†, Fumi Utsumi[1], Hiromasa Tanaka [2, 3], Masaaki Mizuno[3], Shinya Toyokuni[4], Masaru Hori [2], Fumitaka Kikkawa [1] and Hiroaki Kajiyama[1]*

†: equally contributed

*: correspondence to Hiroaki Kajiyama (kajiyama@med.nagoya-u.ac.jp)

[1] Department of Obstetrics and Gynecology, Nagoya University Graduate School of Medicine, Tsurumai-cho 65, Showa-ku, Nagoya 466-8550, Japan
[2] Institute of Innovation for Future Society, Nagoya University, Furo-cho, Chikusa-ku, Nagoya 464-8603, Japan
[3] Center for Advanced Medicine and Clinical Research, Nagoya University Hospital, Tsurumai-cho 65, Showa-ku, Nagoya 466-8550, Japan
[4] Department of Pathology and Biological Responses, Nagoya University Graduate School of Medicine, Tsurumai-cho 65, Showa-ku, Nagoya 466-8550, Japan

Supplementary Figure 1: Migration assay by a real-time cell imaging system

To assess migration, a real-time cell imaging system (IncuCyte™, Essen BioScience Inc., Ann Arbor, MI, USA) was used in accordance with the manufacturer's instructions. 2×10^4 ES2 cells were seeded into each well of 96-well ImageLock tissue culture plates (Essen BioScience) and incubated for 18 h. The wells with the cells were wounded by using the 96-pin WoundMaker™ (Essen BioScience) and immediately exposed to diluted PAM for 30 min. After treatment, the wells were replaced to culture medium and incubated in regular culture conditions. Wound images were automatically recorded 1 h intervals for 12 h and analyzed by the IncuCyte™ software system (Essen BioScience). The data were provided as Relative Wound Density, means ± SD. Sequential photographs of control-PAM and PAM (dilution ratios of 1:64 and 1:8) treated wells were edited for video files (video 1, 2 and 3, respectively).

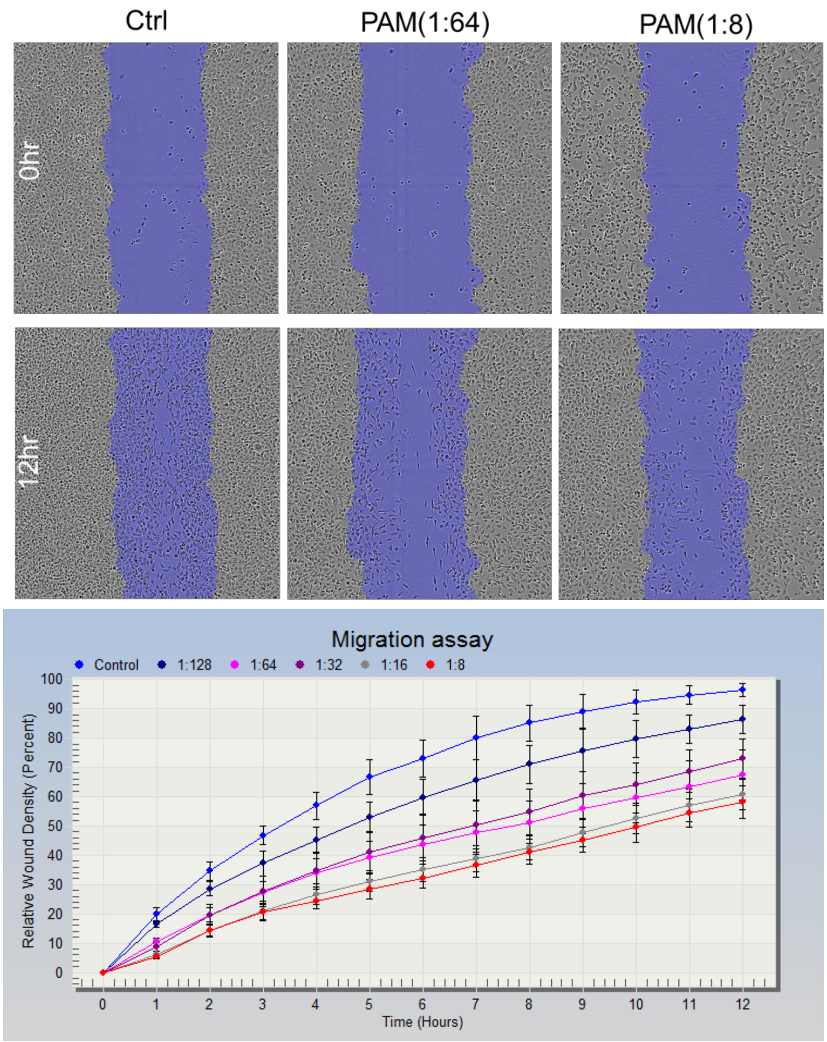

Supplementary Figure 2: Invasion assay by a real-time cell imaging system

For invasion assay, 2.5 × 10^4 ES2 cells were seeded into wells of 96-well ImageLock plates pre-coated with matrigel (100μg/mL). On the following day, wounds were made using the same system of migration assay. The cells were then treated with diluted PAM for 30 min, subsequently overloaded with matrigel (3mg/mL) and incubated at 37 °C for 30 min, constructing a 3D matrix. Growth medium was added on top of the 3D matrix. Wound images were continuously recorded 1 h intervals for 36 h and analyzed by the IncuCyte^{TM} software system (Essen BioScience). The data were provided as Relative Wound density, means ± SD. Sequential photographs of control-PAM and PAM (dilution ratios of 1:64 and 1:8) treated wells were edited for video files (video 4, 5 and 6, respectively).

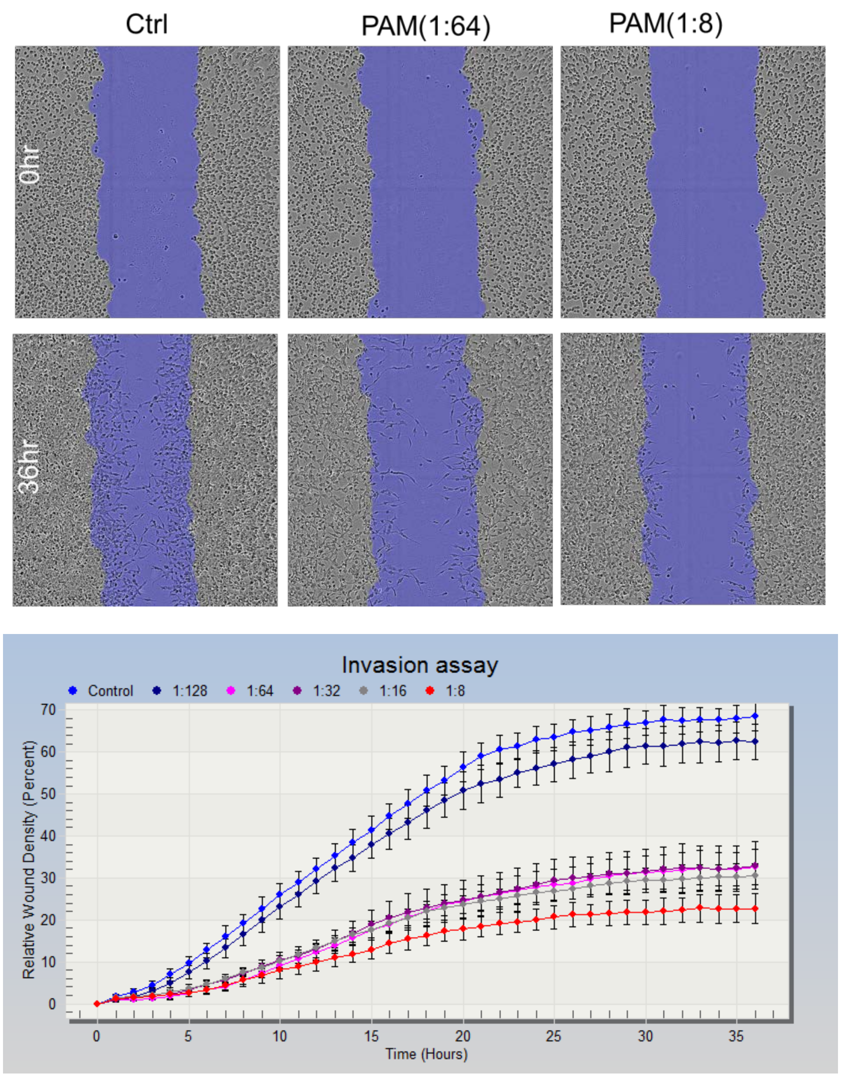

Supplementary Figure 3. The original full-length blots in the manuscript are presented.

To accurately separate and better identify targeting bands, ***Thermo Scientific PageRuler Prestained Protein ladder* (#26616)** was purchased and used in this study. Separated locations of protein ladder were marked in the right side of each blot.

Fig. 1F

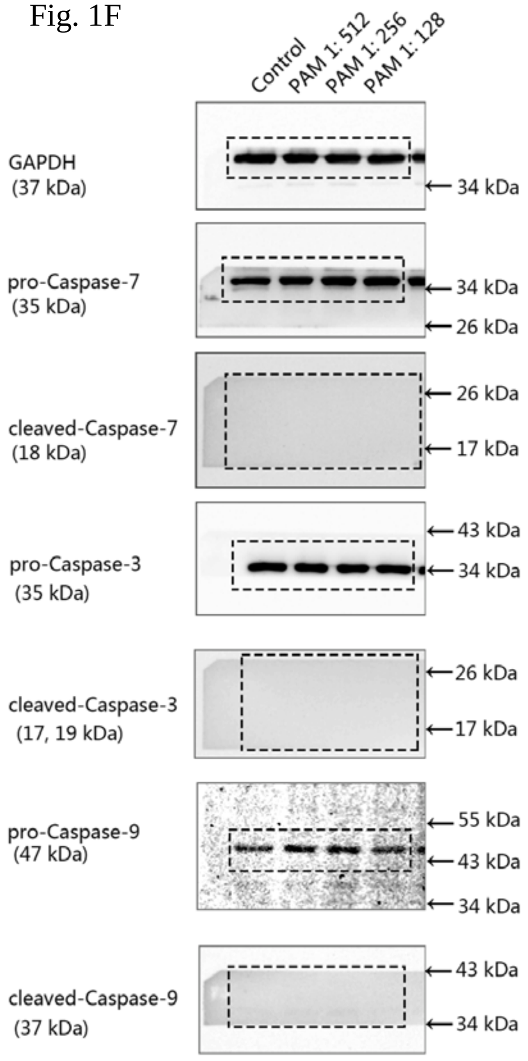

Fig. 4B

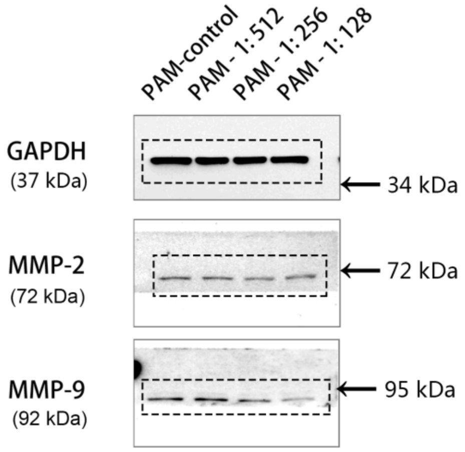

Fig. 5A

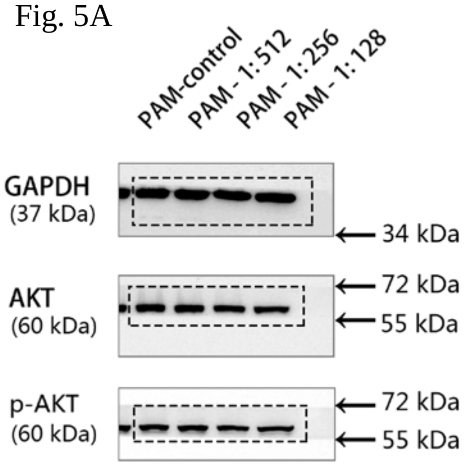
PAM-control
PAM - 1:512
PAM - 1:256
PAM - 1:128
GAPDH
(37 kDa)
34 kDa
AKT
(60 kDa)
72 kDa
55 kDa
p-AKT
(60 kDa)
72 kDa
55 kDa

Fig. 5B

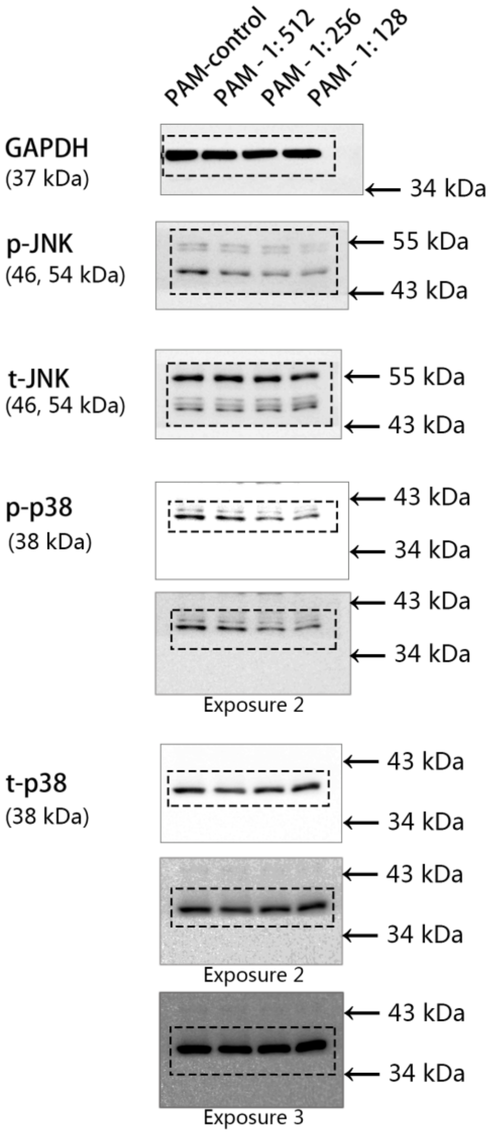
PAM-control
PAM - 1:512
PAM - 1:256
PAM - 1:128
GAPDH
(37 kDa)
34 kDa
p-JNK
(46, 54 kDa)
55 kDa
43 kDa
t-JNK
(46, 54 kDa)
55 kDa
43 kDa
p-p38
(38 kDa)
43 kDa
34 kDa
43 kDa
34 kDa
Exposure 2
t-p38
(38 kDa)
43 kDa
34 kDa
43 kDa
34 kDa
Exposure 2
43 kDa
34 kDa
Exposure 3

Fig. 6C

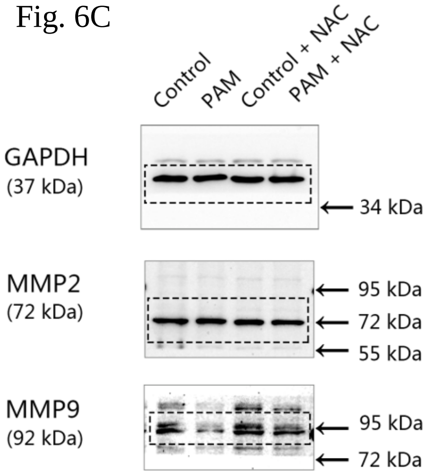
Control
PAM
Control + NAC
PAM + NAC
GAPDH
(37 kDa)
34 kDa
MMP2
(72 kDa)
95 kDa
72 kDa
55 kDa
MMP9
(92 kDa)
95 kDa
72 kDa

Fig. 6D

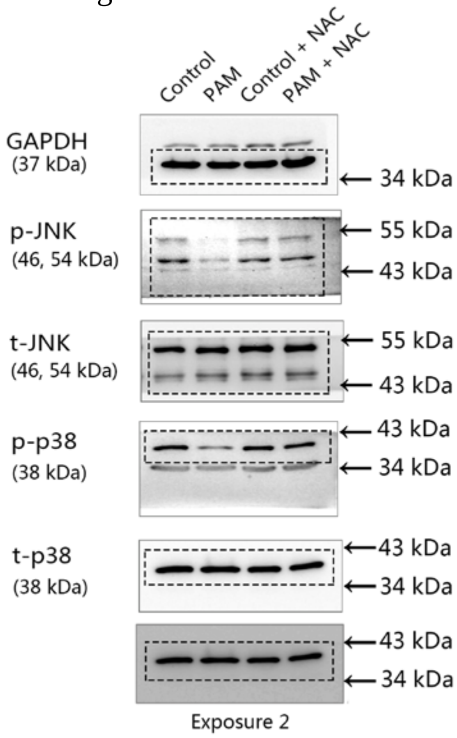
Control
PAM
Control + NAC
PAM + NAC
GAPDH
(37 kDa)
34 kDa
p-JNK
(46, 54 kDa)
55 kDa
43 kDa
t-JNK
(46, 54 kDa)
55 kDa
43 kDa
p-p38
(38 kDa)
43 kDa
34 kDa
t-p38
(38 kDa)
43 kDa
34 kDa
43 kDa
34 kDa
Exposure 2
